# Fração de Ejeção Intermediária na Insuficiência Cardíaca

**DOI:** 10.36660/abc.20200576

**Published:** 2021-01-27

**Authors:** Paula Felippe Martinez, Marina Politi Okoshi, Katashi Okoshi, Silvio Assis de Oliveira-Junior

**Affiliations:** 1 Instituto Integrado de Saúde Universidade Federal de Mato Grosso do Sul Campo GrandeMS Brasil Instituto Integrado de Saúde, Universidade Federal de Mato Grosso do Sul (UFMS), Campo Grande, MS - Brasil; 2 Departamento de Clínica Médica Faculdade de Medicina de Botucatu Universidade Estadual Paulista Júlio de Mesquita Filho BotucatuSP Brasil Departamento de Clínica Médica, Faculdade de Medicina de Botucatu, Universidade Estadual Paulista Júlio de Mesquita Filho (UNESP), Botucatu, SP – Brasil

**Keywords:** Insuficiência Cardíaca/mortalidade, Volume Sistólico, Prognóstico, Adesão a Medicação

A insuficiência cardíaca (IC) é uma síndrome clínica com sintomas típicos causados por anormalidades cardíacas estruturais e/ou funcionais. Sua prevalência é de até 1-2% em adultos de países desenvolvidos com alta mortalidade por causas cardiovasculares.^1 , 2^ A taxa de morbimortalidade também é elevada em países em desenvolvimento como o Brasil.^3^

A principal terminologia usada para classificar a IC se baseia nos valores da fração de ejeção do ventrículo esquerdo (FEVE). Em 2016, a Força-Tarefa para o Diagnóstico e Tratamento da Insuficiência Cardíaca Aguda e Crônica da *European Society of Cardiology* (ESC) introduziu uma nova classe de IC composta por pacientes com FEVE entre 40 e 49%, que foi chamada de IC com fração de ejeção intermediária (ICFEi).^1^ Uma área de indefinição entre insuficiência cardíaca com fração de ejeção reduzida (ICFEr) e preservada (ICFEp) foi reconhecida em estudos anteriores. A introdução dessa nova classificação de IC levou a um rápido aumento no número de estudos sobre ICFEi nos anos seguintes,^4 , 5^ com muitos resultados conflitantes em termos de sobrevida e características clínicas de ICFEi. Embora a mortalidade e a morbidade na ICFEr tenham sido reduzidas com as melhorias nos tratamentos nos últimos 30 anos, os resultados não foram semelhantes para a ICFEp e poucos estudos foram especificamente desenhados para avaliar a mortalidade em pacientes com ICFEi.^6^

Nesta edição dos Arquivos Brasileiros de Cardiologia, lemos com grande interesse o estudo de Petersen et al.^7^ sobre as características clínicas e a taxa de sobrevida de pacientes com IC, comparando ICFEi com fração de ejeção reduzida e preservada. O estudo de coorte acompanhou 380 pacientes adultos com IC aguda internados no pronto atendimento para cardiologia em um hospital terciário de referência no Sul do Brasil. Curiosamente, os pacientes com ICFEi apresentaram características intermediárias de idade, pressão arterial e diâmetro ventricular entre ICFEp e ICFEr. A maioria dos pacientes com ICFEi tinha hipertensão arterial e isquemia miocárdica. Embora a curva de Kaplan-Meier não tenha mostrado diferenças na taxa de sobrevida global entre os diferentes grupos de fração de ejeção, a mortalidade por causas cardiovasculares foi maior em pacientes com ICFEi do que em pacientes com ICFEp, e menor comparada a pacientes com ICFEr. O estudo teve o potencial de uma amostra de tamanho considerável e um tempo médio de acompanhamento mais longo, de aproximadamente quatro anos.

Os resultados desse estudo estão de acordo com pesquisas observacionais anteriores e registros clínicos, que mostraram que os pacientes com ICFEi geralmente apresentam um perfil clínico intermediário, entre a FEVE preservada e reduzida.^8^ No entanto, o prognóstico dos pacientes com ICFEi ainda está em discussão, principalmente considerando que a FEVE muda ao longo do tempo, levantando a questão sobre o estado transicional de ICFEi entre ICFEp e ICFEr.^8^ Uma avaliação longitudinal da FEVE usando o *Swedish Heart Failure Registry* mostrou que pacientes com ICFEi mudaram para ICFEp, ICFEr ou permaneceram no mesmo estágio aproximadamente nas mesmas proporções.^8 , 9^ Além disso, estudos recentes mostraram taxas de eventos reduzidas ou semelhantes em ICFEi em comparação com ICFEr.^8^

Os prós e contras de uma classificação baseada na FEVE para pacientes com ICFEi foram discutidos recentemente.^8^ O uso de outros parâmetros ecocardiográficos, incluindo uma avaliação detalhada das funções sistólica e diastólica, pode ajudar a definir melhor o fenótipo e o prognóstico dos pacientes com ICFEi. Em um modelo experimental de longo prazo, usando uma combinação de parâmetros estruturais cardíacos e ecocardiográficos sistólicos e diastólicos do VE, foi possível diagnosticar de forma não invasiva a IC em ratos após um infarto.^10^ Inclusão de variáveis adicionais, como outros parâmetros de imagem e biomarcadores, etiologia da IC, idade e comorbidades para caracterizar pacientes com IC devem melhorar a compreensão na área de indefinição da ICFEi.^11^
