# Supplementary material for: Fração de Ejeção Intermediária na Insuficiência Cardíaca
Source: Arq Bras Cardiol. 2021 Jan 27;116(1):24–5. [Article in Portuguese] doi: 10.36660/abc.20200576 (PMC8159510; doi:10.36660/abc.20200576)
Supplement: Translation

# Translation: Heart Failure Mid-Range Ejection Fraction

**Authors:** Paula Felippe Martinez, Marina Politi Okoshi, Katashi Okoshi, Silvio Assis de Oliveira-Junior

**Affiliations:** 1 Brazil Integrated Institute of Health, Federal University of Mato Grosso do Sul (UFMS), Campo Grande, MS - Brazil; 2 Brazil Internal Medicine Department, Botucatu Medical School, Sao Paulo State University (UNESP), Botucatu, SP – Brazil

**Keywords:** Heart Failure/mortality, Stroke Volume, Prognostic, Medication, Adherence

Heart failure (HF) is a clinical syndrome with typical symptoms caused by structural and/or functional cardiac abnormalities. It has a prevalence of up to 1-2% in adults from developed countries with high mortality due to cardiovascular causes.^1 , 2^Elevated morbidity and mortality can also be seen in developing countries such as Brazil.^3^

The main terminology used to classify HF is based on left ventricular ejection fraction (LVEF) values. In 2016, the European Society of Cardiology (ESC) Task Force for the Diagnosis and Treatment of Acute and Chronic Heart Failure introduced a new HF class consisting of patients with an LVEF between 40 and 49%, which was called HF with mid-range ejection fraction (HFmrEF).^1^A grey area between heart failure with reduced (HFrEF) and preserved (HFpEF) ejection fraction had been recognized in previous studies. The introduction of this new HF classification led to a rapid increase in the number of studies on HFmrEF over the next few years,^4 , 5^with many conflicting results in terms of survival and the clinical characteristics of HFmrEF being reported in literature. Although mortality and morbidity in HFrEF has been reduced by improving treatment in the last thirty years, similar results were not seen in HFpEF and few studies were specifically designed to evaluate mortality in patients with HFmrEF.^6^

In the current edition of *Arquivos Brasileiros de Cardiologia* , we read with great interest the study by Petersen et al.^7^about the clinical characteristics and survival rate of HF patients, comparing HFmrEF with reduced and preserved ejection fraction. The cohort study followed up 380 adult patients with acute HF admitted via the emergency department to cardiology in a reference tertiary hospital in South Brazil. Interestingly, patients with HFmrEF showed intermediate age, blood pressure, and ventricular diameter characteristics between those of HFpEF and HFrEF. Most patients with HFmrEF had arterial hypertension and myocardial ischemia. Although a Kaplan-Meier curve showed no differences in overall survival rate between the different ejection fraction groups, mortality due to a cardiovascular cause was higher in patients with HFmrEF than those with HFpEF, and lower than those with HFrEF. The study had the strength of a considerable sample size and a long median follow up time of approximately four years.

The results of their study are in accordance with previous observational research and clinical records, which have shown that patients with HFmrEF usually present an intermediate clinical profile between preserved and reduced LVEF.^8^However, the prognosis of HFmrEF patients is still a matter of discussion, particularly considering that LVEF changes over time, raising the question about the transitional status of HFmrEF between HFpEF and HFrEF.^8^A longitudinal evaluation of LVEF using the Swedish Heart Failure Registry showed that HFmrEF patients moved to HFpEF, HFrEF, or remained as HFmrEF in approximately the same proportions.^8 , 9^Furthermore, recent studies have shown both reduced or similar event rates in HFmrEF compared to HFrEF.^8^

The pros and cons of an LVEF-based classification for patients with HFmrEF have recently been discussed.^8^The use of other echocardiographic parameters including a detailed evaluation of systolic and diastolic function could help to better define the phenotype and prognosis of patients with HFmrEF. In a long-term experimental model, by using a combination of cardiac structural and echocardiographic LV systolic and diastolic functional parameters, it was possible to non-invasively diagnose HF in post-infarction rats.^10^The inclusion of additional variables such as other imaging parameters and biomarkers, HF etiology, age, and co-morbidities to characterize HF patients should improve understanding in the gray area of HFmrEF.^11^
